# 598 A Sixteen Year Review of Climate Change and Burns Sustained from Contact with the Ground

**DOI:** 10.1093/jbcr/iraf019.227

**Published:** 2025-04-01

**Authors:** Kevin Foster, Claudia Islas, Louis Ferrari, Karen Richey

**Affiliations:** Diane & Bruce Halle Arizona Burn Center; Diane & Bruce Halle Arizona Burn Center; Diane & Bruce Halle Arizona Burn Center; Diane & Bruce Halle Arizona Burn Center

## Abstract

**Introduction:**

The hot summer months of the desert southwest have resulted in burn injuries sustained from contact with the ground (CWG) for decades. However, over the last decade our center has seen a rapid increase in the number of admissions of this subset of contact burns. This corresponds with the Environmental Protection Agency report that globally 2014-2023 was the warmest decade on record. The purpose of this study was to examine the relationship between temperatures, admissions and outcomes of those patients whose injuries were sustained from CWG.

**Methods:**

This was a retrospective review of patients admitted to our center over the last 16 years, during June, July and August who suffered a CWG. Basic demographic, injury and outcome data were collected. Climate data was obtained from the National Oceanic and Atmospheric Administration website. Multivariate linear regression analysis was used to examine the relationship between temperature and Total Body Surface Area (%TBSA). Chi-Square test of independence was performed to assess associations between admissions and temperature. A piecewise linear regression model was applied to identify a threshold temperature where admissions and TBSA exhibited significant change. Logistic regression was used to evaluate predictors of mortality.

**Results:**

A total of 846 CWG admissions were recorded over the review period, with admissions increasing from 9 in 2008 to 120 in 2023. Correspondingly, the average max temperature increased from 106.8 to 112.9 degrees. Chi-square test of independence showed a significant association between these two variables (x2=153.21, p <.001, df=15). Multivariate regression analysis examining the relationship between max temperature and % TBSA revealed that for each 1◦F increase in temperature, %TBSA increased by 1.4% (p <.001). Piecewise linear regression analysis identified a temperature threshold of 105◦F, where admission and %TBSA rose significantly. Patients who died were older (63 vs 52 years, p <.0001). Regression models, showed that for every added year of age, mortality increased by 3.34% (p <.0001) and for each 1% increase in TBSA, mortality increased by 15.09% (p <.001).

**Conclusions:**

Environmental heat is a significant public health problem. The true scope of the problem is difficult to ascertain as deaths that occurred outside of the center are not included in this analysis. CWG patients not only present with thermal injury but often suffer concomitant heat stroke, worsening outcomes. The human toll is not limited to patients, the spike in admissions puts tremendous strain on the center’s resources. As the number of regions experiencing intense, extreme heat expands, so will the number of centers treating this subset of contact burns.

**Applicability of Research to Practice:**

Partnership with community stakeholders is recommended to proactively address heat-related risks and minimize the threat to society.

**Funding for the Study:**

N/A

